# Editorial: Interdisciplinary Approaches to Improve Quality of Soft Fruit Berries

**DOI:** 10.3389/fpls.2020.592222

**Published:** 2020-09-10

**Authors:** Brian Farneti, Francesco Emanuelli, Lara Giongo, Peter Toivonen, Massimo Iorizzo, Kevin Folta, Chad Finn

**Affiliations:** ^1^ Genomics and Biology of Fruit Crop Department, Fondazione Edmund Mach, San Michele all’Adige, Italy; ^2^ Summerland Research and Development Centre, Agriculture and Agri-Food Canada, Summerland, BC, Canada; ^3^ Department of Horticultural Science, North Carolina State University, Raleigh, NC, United States; ^4^ Horticultural Sciences Department, University of Florida, Gainesville, FL, United States; ^5^ Horticultural Crops Research Unit, Agricultural Research Service, United States Department of Agriculture, Corvallis, OR, United States

**Keywords:** quality, breeding, omics analyses, vaccinium, rubus, fragaria, postharvest, nutraceutical

Improving the fruit quality is a chief target to support the rising global economic importance of berry crops (Di Vittori et al., 2018). Quality of soft fruit berries is a complex trait, which includes visual attractiveness (color, size, and shape), overall flavor (taste and texture), and nutritional properties. Among these traits, texture, flavor, and appearance directly impact postharvest performance and consumer appreciation and therefore fruit marketability. Although the importance of these factors can hardly be underestimated, breeding efforts have historically been mainly oriented to improve fruit appearance and storability. However, selection for improved shelf-life and appearance properties, and inappropriate postharvest strategies, may have unintended negative consequences on other fruit quality traits, for instance, aroma and nutritional value. This quality decline can be heightened by the fact that breeding selection for flavor occurs nearly by chance (not assisted), since flavor and nutraceutical content are currently not considered as a discriminating trait in the early selection phase (Klee and Tieman, 2018). This limitation is also reinforced by the fact that complex and time-consuming phenotyping protocols are ordinarily used, making the analytical screening of large populations plant material unrealistic (Folta and Klee, 2016). This Research Topic has aimed to collect the most recent advances on scientific progress concerning the quality of soft fruit berries, in particular strawberry (*Fragaria ananassa*), blueberry (*Vaccinium* spp.), and raspberry (*Rubus idaeus*). These studies focused on innovative technologies and multidisciplinary approaches of quality management throughout the entire production chain, from breeding selection to consumer consumption.

In this article collection authors have discussed the importance of developing chemical and molecular markers to assist breeding selection, in particular for three main features: controlling of the flowering period (Jibran et al.), improving quality and storability (Farneti et al.), and increasing the nutraceutical content (Mengist et al.). The study of Jibran et al. focused on raspberry (*Rubus idaeus*) and blackberry (*Rubus* subgenus *Rubus*) segregating populations and discovered that two major loci (RiAF3 and RiAF4) and a region located on the upper arm of LG7 are controlling the annual-fruiting (AF) trait in *Rubus*. AF varieties of *Rubus* are able to flower and fruit in one growing season, without the occurring dormant period required in biennial-fruiting varieties. Molecular assisted selection of accessions with the AF trait would be beneficial for a more sustainable and schedulable *Rubus* production.

The studies of Farneti et al. and Mengist et al. were mainly focused on discovery the metabolic and quality variability among blueberry (*Vaccinium* spp.) genotypes, to provide a framework to uncover the genetic basis of bioactive compounds and fruit quality traits useful to advance blueberry-breeding programs focusing on integrating these traits. The exploitation of the genetic variability existing within the blueberry germplasm collection allowed Farneti et al. to identify the best performing cultivars to be used as superior parental lines for future breeding programs, based on texture and volatile organic compounds (VOCs) variability. In particular, the comprehensive characterization of blueberry aroma by direct injection mass spectrometry technique allowed the identification of a wide array of VOCs that can be used as putative biomarkers to rapidly evaluate the blueberry aroma variations related to genetic differences and storability.

Mengist et al. assessed the variability of different nutraceutical metabolites among blueberry accessions of three ploidy levels (diploid, tetraploid, and hexaploid). Results of that study revealed a moderate to high broad sense heritability for many metabolites, suggesting a strong role of genetic factors in controlling these traits in blueberry fruit. In addition, despite a relevant fruit size-dependent variation for anthocyanin content, metabolite concentrations and fruit size, to a certain degree, can be improved simultaneously in breeding programs. Regulation of anthocyanin production in blueberry fruit was further investigated by Günther et al. By linking the accumulation patterns of phenolic metabolites with gene transcription in Northern Highbush (*Vaccinium corymbosum*) and Rabbiteye (*Vaccinium virgatum*) blueberry, they found that flavonoid production was generally lower in fruit flesh compared with skin and concentrations further declined during maturation. A common set of structural genes was identified across both species, indicating that tissue-specific flavonoid biosynthesis was dependent on co-expression of multiple pathway genes and limited by the phenylpropanoid pathway in combination with CHS, F3H, and ANS as potential pathway bottlenecks. Moreover, they identified several candidate transcriptional regulators that were co-expressed with structural genes, including the activators MYBA, MYBPA1, and bHLH2 together with the repressor MYBC2, which suggested an interdependent role in anthocyanin regulation.

Anthocyanin content was considerably improved with preharvest agronomic practices in grapevine berry and strawberry in the studies of Pereira et al. and Zuñiga et al., respectively. Pereira et al. found that trunk girdling applied at veraison, in “Cabernet Sauvignon” wine grapes (*Vitis vinifera* L.) increased anthocyanin and flavonol concentrations in skin/pulp tissues of grape berries without affecting primary metabolites. Trunk girdling trials might be applied to change the source-sink relationship on blueberry as already reported by Jorquera-Fontena et al. (2016). Zuñiga et al. demonstrated that increasing the number of methyl jasmonate preharvest applications (maximum three treatments) on strawberry (*Fragaria ananassa* “Camarosa”) improved the anthocyanin, proanthocyanidin, and ascorbic acid content of the fruit, as well as the antioxidant-related enzymatic activity during postharvest storage.

Improving the storability and the acceptance period of berry fruit is of chief importance, being the short commercial life one of the strong limiting factor of berries. Even if fruit storability is often regulated by genetic differences (Farneti et al.) and/or preharvest agronomic practices (Zuniga et al.) optimized postharvest methods are needed. Tosetti et al. elucidated the role of ethylene in strawberry postharvest physiology. Continuous exposure to ethylene induced an accumulation of abscisic acid in the receptacle tissue, followed by an increase in CO_2_ production. Ethylene also elicited sucrose hydrolysis and malic acid catabolism, with the major effect seen after 4 days of ethylene exposure. Additionally, ethylene treatment induced an accumulation of phenolics (epicatechin and chlorogenic acid). Ethylene can intensely affect quality of harvested products. These effects can be beneficial or deleterious depending on the product, its ripening stage, and its desired use. Thus, new strategies for controlling ethylene production are needed. Shu et al. proved that 1-MCP treatment could effectively maintain the quality of the “Laiyang” pear during cold storage, and the additional application of ethephon on fruits during shelf-life managed to restore volatile aromas in pear fruits after long-term storage. Postharvest storability of blueberry, instead, was significantly improved by the development of an innovative controlled atmosphere (CA) approach, proposed by Falagán et al., based on gradually reaching the optimal storage concentrations (GCA). This methodology allowed the reduction of blueberry respiratory metabolism when compared to standard CA and control treatments. This had a positive impact on quality parameters such as sugars, organic acids, firmness and decay incidence.

Understanding the stability of each quality trait during different storage and/or agronomical conditions may allow a better definition of future breeding strategies aimed at the selection of accessions suitable to improve distinct market sector performance. For this purpose, comprehensive investigations and a tight synergy of analytical approaches, from different branches of knowledge, are needed. The expanded use of inexpensive, high-quality, and high throughput omics techniques is expected to soon provide elucidation of the genetic and physiological regulation of fruit quality.

The completion of this work is overshadowed by the tragic loss of one of its co-editors. In his 26 years, Dr. Chad Finn served as a world-renowned berry-crop breeder, contributing to the release of 51 cultivars in blackberry, red and black raspberry, strawberry, blueberry, and other crops as part of the USDA-ARS and Oregon State University. We will miss his internationally respected collegiality and work ethic, his industry-leading cultivars, and professional contributions to the discipline. But most of all, we will miss his kind guidance and mentoring, his booming laugh, and his bone-crushing hugs. We dedicate this Research Topic in Dr. Chad Finn’s memory.

## Author Contributions

All authors listed have made a substantial, direct, and intellectual contribution to the work and approved it for publication.

## Conflict of Interest

The authors declare that the research was conducted in the absence of any commercial or financial relationships that could be construed as a potential conflict of interest.
